# Polymorphism of rs1044925 in the acyl-CoA:cholesterol acyltransferase-1 gene and serum lipid levels in the Guangxi Bai Ku Yao and Han populations

**DOI:** 10.1186/1476-511X-9-139

**Published:** 2010-12-08

**Authors:** Dong-Feng Wu, Rui-Xing Yin, Lynn Htet Htet Aung, Xi-Jiang Hu, Xiao-Li Cao, Lin Miao, Qing Li, Ting-Ting Yan, Jin-Zhen Wu, Shang-Ling Pan

**Affiliations:** 1 Department of Cardiology, Institute of Cardiovascular Diseases, the First Affiliated Hospital, Guangxi Medical University, 22 Shuangyong Road, Nanning 530021, Guangxi, China; 2 Department of Pathophysiology, School of Premedical Sciences, Guangxi Medical University, Nanning 530021, Guangxi, China

## Abstract

**Background:**

The association of rs1044925 polymorphism in the acyl-CoA:cholesterol acyltransferase-1 (ACAT-1) gene and serum lipid profiles is not well known in different ethnic groups. Bai Ku Yao is a special subgroup of the Yao minority in China. The present study was carried out to clarify the association of rs1044925 polymorphism in the ACAT-1 gene and several environmental factors with serum lipid levels in the Guangxi Bai Ku Yao and Han populations.

**Methods:**

A total of 626 subjects of Bai Ku Yao and 624 participants of Han Chinese were randomly selected from our previous stratified randomized cluster samples. Genotyping of rs1044925 polymorphism in the ACAT-1 gene was performed by polymerase chain reaction and restriction fragment length polymorphism combined with gel electrophoresis, and then confirmed by direct sequencing.

**Results:**

The levels of serum total cholesterol (TC), high-density lipoprotein cholesterol (HDL-C), apolipoprotein (Apo) AI and ApoB were lower in Bai Ku Yao than in Han (*P *< 0.01 for all). The frequency of A and C alleles was 79.0% and 21.0% in Bai Ku Yao, and 87.3% and 12.7% in Han (*P *< 0.001); respectively. The frequency of AA, AC and CC genotypes was 63.2%, 31.4% and 5.2% in Bai Ku Yao, and 75.6%, 23.2% and 1.1% in Han (*P *< 0.001); respectively. The levels of TC, LDL-C and ApoB in Bai Ku Yao but not in Han were different between the AA and AC/CC genotypes in females but not in males (*P *< 0.05 for all). The C allele carriers had lower serum TC, LDL-C and ApoB levels as compared with the C allele noncarriers. The levels of TC, LDL-C and ApoB in Bai Ku Yao but not in Han were correlated with genotypes in females but not in males (*P *< 0.05 for all). Serum lipid parameters were also correlated with sex, age, body mass index, alcohol consumption, and blood pressure in both ethnic groups (*P *< 0.05-0.001).

**Conclusions:**

These results suggest that the polymorphism of rs1044925 in the ACAT-1 gene is mainly associated with female serum TC, LDL-C and ApoB levels in the Bai Ku Yao population. The C allele carriers had lower serum TC, LDL-C and ApoB levels than the C allele noncarriers.

## Introduction

Coronary artery disease (CAD) is a major health problem and the leading cause of death in many industrialized countries [1,2]. Dyslipidemia such as elevated serum levels of total cholesterol (TC) [3], triglyceride (TG) [4], low-density lipoprotein cholesterol (LDL-C) [5], and apolipoprotein (Apo) B [6], or low levels of high-density lipoprotein cholesterol (HDL-C) and ApoAI [6-8] is one of the most important modifiable risk factors for CAD in western [9,10] as well as in Asian [11,12] populations. It has been well established that dyslipidemia is a complex trait caused by multiple environmental [13,14] and genetic factors [15-17] and their interactions [18,19]. Family history and twin studies have shown that genetic polymorphism could account for 40-60% of the interindividual variation in plasma lipid phenotypes [20-22].

Cholesterol in the body is present in tissues and plasma lipoproteins either as free cholesterol or cholesteryl esters. The esterification of cholesterol in plasma by lecithin: cholesterol acyltransferase (LCAT) plays an important role in the intravascular maturation of lipoproteins, sepecially HDL. In contrast, intracellular cholesteryl ester synthesis, catalyzed by acyl-CoA:cholesterol acyltransferase (ACAT, E.C.2.3.1.26), also called sterol *o*-acyltransferase (SOAT), serves to store cholesterol in cytosolic droplets and also participates in the hepatic secretion of lipoproteins containing ApoB [23-26]. ACAT has found to be present as two isoforms, ACAT-1 and ACAT-2, with different intracellular localizations, membrane topology in mammalian species, and metabolic function for each enzyme [27-29]. ACAT-1 is ubiquitously expressed in various tissues and cells including adrenal glands, kidney [30-32], and macrophages [33] and is responsible for foam cell formation in macrophages, whereas ACAT-2 is expressed only in intestine and liver [27,28,34] and is in charge of the cholesterol absorption process in intestinal mucosal cells [35]. The first ACAT-1 cDNA was cloned by Chang and colleagues [30] in 1993. ACAT-2 was cloned in 1998 [27,28,34]. The two genes have 47% overall nucleotide identity [30,34] and encode specific ACAT proteins that have 43% amino acid sequence identity and 63% similarity. ACAT activity has been detected in a diverse range of tissues and cell types [36] and is known to play an important role in cell biology and in the pathogenesis of important lipid-related diseases such as atherosclerosis [36] and cholesterol gallstones [37]. For example, ACAT has been shown to play a pivotal functional role in the intestinal absorption of cholesterol, the hepatic secretion of very low-density lipoprotein (VLDL), the biosynthesis of steroid hormones, the production of cholesteryl esters in macrophages (foam cells) in atheroma, and the secretion of biliary cholesterol. From a biochemical and physiological perspective, ACAT is one of the central enzymes regulating plasma and biliary cholesterol concentrations. In several previous studies, two single nucleotide polymorphisms (SNPs) of rs1044925 and -77G > A in the ACAT-1 gene were associated with modifications of serum lipid concentrations [38,39] and with low cerebrospinal fluid levels of cholesterol [40], but one SNP (R526G) in the ACAT-1 gene was not associated with serum lipid parameters [39].

China is a vast and diverse country. There are 56 ethnic groups. Han is the largest ethnic group and Yao is the eleventh largest minority among the 55 minority groups according to the population size. Bai Ku Yao (White-trouser Yao), an isolated branch of the Yao minority, is named so because all of men wear white knee-length knickerbockers. The population size is about 30000. Because of isolation from the other ethnic groups, the special customs and cultures including their clothing, intra-ethnic marriages, ballad, funeral, bronze drum, spinning top, dietary habits, and corn wine and rum intakes are still completely preserved to the present day. They are currently in a transitional period from the matriarchal society to patriarchal society. In several previous epidemiological studies, we showed that several serum lipid phenotypes were lower in Bai Ku Yao than in Han Chinese from the same region [13,14]. This ethnic difference in serum lipid profiles is still not well known. We hypothesized that some genetic factors may be different between the two ethnic groups. Therefore, the aim of the present study was to detect the association of rs1044925 polymorphism in the ACAT-1 gene and several environmental factors with serum lipid phenotypes in the Guangxi Bai Ku Yao and Han populations.

## Materials and methods

### Study population

A total of 626 subjects of Bai Ku Yao who reside in Lihu and Baxu villages in Nandan County, Guangxi Zhuang Autonomous Region, People's Republic of China were randomly selected from our previous stratified randomized cluster samples [13,14]. The ages of the subjects ranged from 15 to 80 years, with an average age of 40.25 ± 14.97 years. There were 305 males (48.72%) and 321 females (51.28%). All subjects were rural agricultural workers. The subjects accounted for 2.09% of total Bai Ku Yao population. During the same period, a total of 624 people of Han Chinese who reside in the same villages were also randomly selected from our previous stratified randomized cluster samples [13,14]. The average age of the subjects was 40.71 ± 15.71 years (range 15 to 80). There were 304 men (48.72%) and 320 women (51.28%). All of them were also rural agricultural workers. All study subjects were essentially healthy and had no evidence of any chronic illness, including hepatic, renal, or thyroid. The participants with a history of heart attack or myocardial infarction, stroke, congestive heart failure, diabetes or fasting blood glucose ≥7.0 mmol/L determined by glucose meter have been excluded. The participants were not taking medications known to affect serum lipid levels (lipid-lowering drugs such as statins or fibrates, beta-blockers, diuretics, or hormones). The present study was approved by the Ethics Committee of the First Affiliated Hospital, Guangxi Medical University. Informed consent was obtained from all subjects after they received a full explanation of the study.

### Epidemiological survey

The survey was carried out using internationally standardized methods, following a common protocol [41]. Information on demographics, socioeconomic status, and lifestyle factors was collected with standardized questionnaires. The alcohol information included questions about the number of liangs (about 50 g) of rice wine, corn wine, rum, beer, or liquor consumed during the preceding 12 months. Alcohol consumption was categorized into groups of grams of alcohol per day: <25 and ≥25. Smoking status was categorized into groups of cigarettes per day: <20 and ≥20. At the physical examination, several anthropometric parameters, such as height, weight, and waist circumference were measured. Sitting blood pressure was measured three times with the use of a mercury sphygmomanometer after the subjects had a 5-minute rest, and the average of the three measurements was used for the level of blood pressure. Systolic blood pressure was determined by the first Korotkoff sound, and diastolic blood pressure by the fifth Korotkoff sound. Body weight, to the nearest 50 grams, was measured using a portable balance scale. Subjects were weighed without shoes and in a minimum of clothing. Height was measured, to the nearest 0.5 cm, using a portable steel measuring device. From these two measurements body mass index (BMI, kg/m^2^) was calculated.

### Biochemical analysis

A venous blood sample of 8 mL was obtained from all subjects between 8 and 11 AM, after at least 12 hours of fasting, from a forearm vein after venous occlusion for few seconds in a sitting position. A part of the sample (3 mL) was collected into glass tubes and allowed to clot at room temperature, and used to determine serum lipid levels. Another part of the sample (5 mL) was transferred to tubes with anticoagulate solution (4.80 g/L citric acid, 14.70 g/L glucose, and 13.20 g/L tri-sodium citrate) and used to extract DNA. Immediately following clotting serum was separated by centrifugation for 15 minutes at 3000 rpm. The levels of TC, TG, HDL-C, and LDL-C in samples were determined by enzymatic methods with commercially available kits, Tcho-1, TG-LH (RANDOX Laboratories Ltd., Ardmore, Diamond Road, Crumlin Co. Antrim, United Kingdom, BT29 4QY), Cholestest N HDL, and Cholestest LDL (Daiichi Pure Chemicals Co., Ltd., Tokyo, Japan); respectively. Serum ApoAI and ApoB levels were detected by the immunoturbidimetric immunoassay using a commercial kit (RANDOX Laboratories Ltd.). All determinations were performed with an autoanalyzer (Type 7170A; Hitachi Ltd., Tokyo, Japan) in the Clinical Science Experiment Center of the First Affiliated Hospital, Guangxi Medical University [13,14].

### DNA amplification and genotyping

Genomic DNA was isolated from peripheral blood leukocytes using the phenol-chloroform method [15-19]. The extracted DNA was stored at 4°C until analysis. Genotyping of the polymorphism of rs1044925 was performed by polymerase chain reaction and restriction fragment length polymorphism (PCR-RFLP) [38,42]. PCR amplification was performed using 5'-TATATTAAGGGGATCAGAAGT-3' and 5'-CCACCTAAAAACATACTACC-3' (Sangon, Shanghai, People's Republic of China) as the forward and reverse primer pairs; respectively. Each amplification reaction was performed using 0.5 μg of genomic DNA in 25 μL of reaction mixture consisting of 0.2 μM of each primer, 200 μM of each deoxynucleotide triphoisphate, 2.5 μL of 10 × PCR buffer (100 mM Tris-HCl, pH 8.3, 500 mM KCl, 20 mM MgCl_2_, 1% Triton), and 1.25 units of *Taq *polymerase. After initial denaturizing at 95°C for 5 min, the reaction mixture was subjected to 33 cycles of 45 s denaturation at 95°C, 30 s annealing at 53°C and extension 50 s at 72°C, followed by a final 10 min extension at 72°C. After electrophoresis on a 1.5% agarose gel with 0.5 μg/mL ethidium bromide, the amplification products were visualized under ultraviolet light. Then 5 U of *Rsa*I restriction enzyme was added directly to the PCR products (5 μL) and digested at 37°C overnight. After restriction enzyme digestion of the amplified DNA, genotypes were identified by electrophoresis on 1.5% agarose gels and visualized with ethidium-bromide staining ultraviolet illumination. The genotypes were scored by an experienced reader blinded to the epidemiological data and serum lipid levels. Six samples (AA, AC and CC genotypes in two; respectively) detected by the PCR-RFLP were also confirmed by direct sequencing. The PCR products were purified by low melting point gel electrophoresis and phenol extraction, and then the DNA sequences were analyzed in Shanghai Sangon Biological Engineering Technology & Services Co., Ltd., People's Republic of China.

### Diagnostic criteria

The normal values of serum TC, TG, HDL-C, LDL-C, ApoAI, ApoB levels, and the ratio of ApoAI to ApoB in our Clinical Science Experiment Center were 3.10-5.17, 0.56-1.70, 0.91-1.81, 2.70-3.20 mmol/L, 1.00-1.78, 0.63-1.14 g/L, and 1.00-2.50; respectively. The individuals with TC > 5.17 mmol/L and/or TG > 1.70 mmol/L were defined as hyperlipidemic [13,14]. Hypertension was diagnosed according to the criteria of 1999 World Health Organization-International Society of Hypertension Guidelines for the management of hypertension [43,44]. The diagnostic criteria of overweight and obesity were according to the Cooperative Meta-analysis Group of China Obesity Task Force. Normal weight, overweight and obesity were defined as a BMI < 24, 24-28, and > 28 kg/m^2^; respectively [45].

### Statistical analyses

Epidemiological data were recorded on a pre-designed form and managed with Excel software. All statistical analyses were done with the statistical software package SPSS 13.0 (SPSS Inc., Chicago, Illinois). Quantitative variables were expressed as mean ± standard deviation (serum TG levels were presented as medians and interquartile ranges). Qualitative variables were expressed as percentages. Allele frequency was determined via direct counting, and the standard goodness-of-fit test was used to test the Hardy-Weinberg equilibrium. Difference in genotype distribution between the groups was obtained using the chi-square test. The difference in general characteristics between Bai Ku Yao and Han was tested by the Student's unpaired *t*-test. The association of genotypes and serum lipid parameters was tested by analysis of covariance (ANCOVA). Sex, age, BMI, blood pressure, alcohol consumption, cigarette smoking were adjusted for the statistical analysis. In order to evaluate the association of serum lipid levels with genotypes (AA = 1, AC/CC = 2) and several environment factors, multiple linear regression analysis with stepwise modeling was also performed in the combined population of Bai Ku Yao and Han, Bai Ku Yao, Han, males and females; respectively. A *P *value of less than 0.05 was considered statistically significant.

## Results

### General characteristics and serum lipid levels

Table 1 gives the general characteristics and serum lipid levels between the Bai Ku Yao and Han populations. The levels of height, weight, BMI, systolic blood pressure, pulse pressure, serum TC, HDL-C, ApoAI, ApoB were lower in Bai Ku Yao than in Han Chinese (*P *< 0.05-0.001), whereas the percentage of subjects who consumed alcohol was higher in Bai Ku Yao than in Han (*P *< 0.001). There was no significant difference in the levels of diastolic blood pressure, serum TG, LDL-C, the ratio of ApoAI to ApoB, age structure, the percentage of subjects who smoked cigarettes, or the ratio of male to female between the two ethnic groups (*P *> 0.05 for all).

**Table 1:** The general characterisrics and serum lipid levels between Bai Ku Yao and Han Chinese

Parameter	Bai Ku Yao	Han Chinese	*t *(χ^2^)	*P*
Number	626	624	-	-
Male/female	305/321	304/320	0.000	0.999
Age (years)	40.25 ± 14.97	40.71 ± 15.71	-0.523	0.601
Height (cm)	152.69 ± 7.46	155.41 ± 8.23	-6.102	0.000
Weight (kg)	51.84 ± 7.17	54.51 ± 9.28	-5.703	0.000
Body mass index (kg/m^2^)	22.20 ± 2.38	22.54 ± 3.22	-2.126	0.034
Systolic blood pressure (mmHg)	119.30 ± 16.97	121.28 ± 16.74	-2.077	0.038
Diastolic blood pressure (mmHg)	75.59 ± 9.53	76.21 ± 10.61	-1.087	0.277
Pulse pressure (mmHg)	43.71 ± 12.83	45.09 ± 11.65	-1.982	0.048
Cigarette smoking [n (%)]				
Nonsmoker	429 (68.5)	448 (71.8)		
< 20 cigarettes/day	90 (14.4)	70 (11.2)		
≥ 20 cigarettes/day	107 (17.1)	106 (17.0)	2.913	0.233
Alcohol consumption [n (%)]				
Nondrinker	334 (53.4)	392 (62.8)		
< 25 g/day	189 (30.2)	160 (25.6)		
≥ 25 g/day	103 (16.5)	72 (11.5)	12.532	0.002
Total cholesterol (mmol/L)	4.34 ± 0.87	4.74 ± 1.02	-7.419	0.000
Triglyceride (mmol/L)	1.00 (0.65)	1.01 (0.69)	-0.982	0.326
HDL-C (mmol/L)	1.66 ± 0.41	1.89 ± 0.50	-8.790	0.000
LDL-C (mmol/L)	2.56 ± 0.72	2.63 ± 0.77	-1.539	0.124
Apolipoprotein (Apo) AI (g/L)	1.31 ± 0.32	1.42 ± 0.28	-6.478	0.000
ApoB (g/L)	0.85 ± 0.22	0.89 ± 0.22	-3.361	0.001
ApoAI/ApoB	1.66 ± 0.72	1.69 ± 0.58	-0.651	0.515

HDL-C, highdensity lipoprotein cholesterol; LDL-C, low-density lipoprotein cholesterol. The value of TG was presented as median (interquartile range). The difference between the two ethnic groups was determined by the Wilcoxon-Mann-Whitney test.

### Results of electrophoresis and genotyping

**Figure 1:**
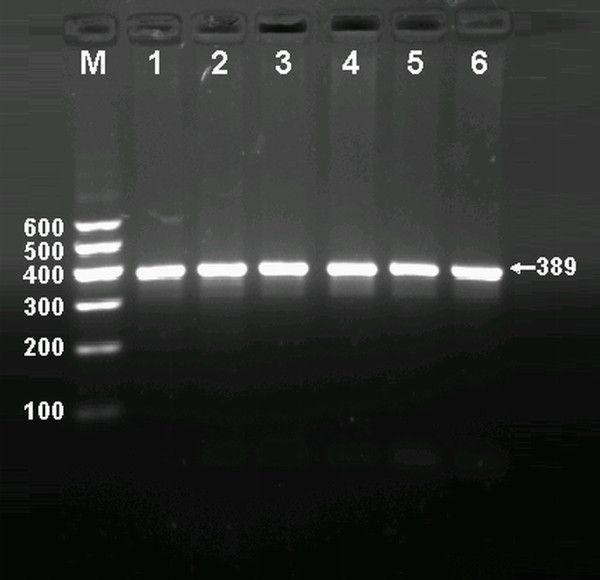
**Electrophoresis of PCR products of the samples**. Lane M, 100 bp marker ladder; lanes 1-6, samples. The 389 bp bands are the target genes.

**Figure 2:**
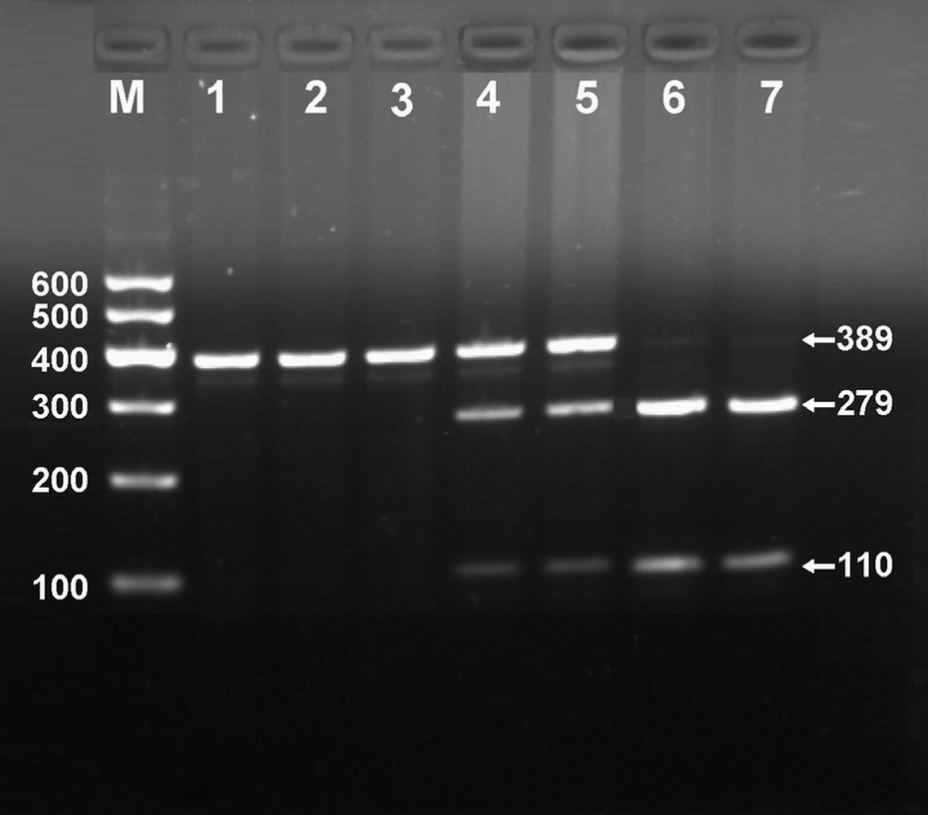
**Genotyping of rs1044925 polymorphism in the ACAT-1 gene**. Lane M, 100 bp marker ladder; lanes 1-3, AA genotype (389 bp); lanes 4 and 5, AC genotype (389-, 279- and 110-bp); and lanes 6 and 7, CC genotype (279- and 110-bp).

After the genomic DNA of the samples was amplified by PCR and imaged by 1.5% agarose gel electrophoresis, the purpose gene of 389 bp nucleotide sequences could be found in all samples (Figure 1). The genotypes identified were named according to the presence or absence of the enzyme restriction sites, when an A to C transversion at rs1044925 locus of the ACAT-1 gene. The presence of the cutting site indicates the C allele, while its absence indicates the A allele (cannot be cut). Thus, the AA genotype is homozygote for the absence of the site (band at 389 bp), AC genotype is heterozygote for the absence and presence of the site (bands at 389-, 279- and 110-bp), and CC genotype is homozygote for the presence of the site (bands at 279- and 110-bp; Figure 2).

### Genotypic and allelic frequencies

The genotypic and allelic frequencies of rs1044925 polymorphism in the ACAT-1 gene are shown in Table 2. The frequency of A and C alleles was 79.0% and 21.0% in Bai Ku Yao, and 87.3% and 12.7% in Han (*P *< 0.001); respectively. The frequency of AA, AC and CC genotypes was 63.2%, 31.4% and 5.2% in Bai Ku Yao, and 75.6%, 23.2% and 1.1% in Han (*P *< 0.001); respectively. There was no significant difference in the genotypic and allelic frequencies between the males and females in both ethnic groups.

**Table 2:** The genotypic and allelic frequencies of rs1044925 polymorphism in the ACAT-1 gene between Bai Ku Yao and Han Chinese [n (%)]

		Genotype			Allele	
			
Group	n	AA	AC	CC	A	C
Bai Ku Yao	626	396 (63.2)	197 (31.4)	33 (5.2)	989 (79.0)	263 (21.0)
Han Chinese	624	472 (75.6)	145 (23.2)	7 (1.1)	1089 (87.3)	159 (12.7)
χ^2^	-	31.458			30.436	
*P*	-	0.000			0.000	
Bai Ku Yao						
Male	305	191 (62.6)	96 (31.5)	18 (5.9)	478 (78.4)	132 (21.6)
Female	321	205 (63.9)	101 (31.5)	15 (4.7)	511 (79.6)	131 (20.4)
χ^2^	-	0.486			0.287	
*P*	-	0.784			0.592	
Han Chinese						
Male	304	223 (73.4)	78 (25.7)	3 (1.0)	524 (86.2)	84 (13.8)
Female	320	249 (77.8)	67 (20.9)	4 (1.3)	565 (88.3)	75 (11.7)
χ^2^	-	2.001			1.233	
*P*	-	0.368			0.267	

### Results of sequencing

**Figure 3:**
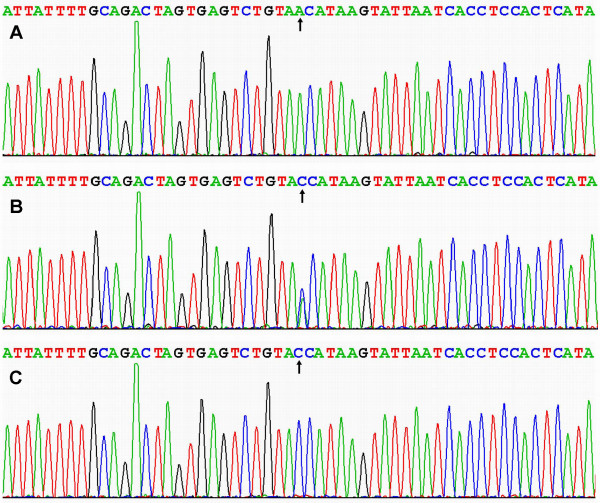
**A part of the nucleotide sequence of rs1044925 in the ACAT-1 gene**. (A) AA genotype; (B) AC genotype; (C) CC genotype.

The results were shown as AA, AC and CC genotypes by PCR-RFLP, the AA, AC and CC genotypes were also confirmed by sequencing (Figure 3); respectively.

### Genotypes and serum lipid levels

As shown in Table 3 the levels of TC, LDL-C and ApoB in Bai Ku Yao but not in Han were significant differences between the AA and AC/CC genotypes in females but not in males (*P *< 0.05 for all). The subjects with AC/CC genotype had lower serum TC, LDL-C and ApoB levels than the subjects with AA genotype. There was no significant difference in serum lipid parameters between the AA and AC/CC genotypes in both ethnic groups (*P *> 0.05 for all).

**Table 3:** The genotypes of rs1044925 polymorphism in the ACAT-1 gene and serum lipid levels between Bai Ku Yao and Han Chinese

Genotype	n	TC (mmol/L)	TG (mmol/L)	HDL-C (mmol/L)	LDL-C (mmol/L)	ApoAI (g/L)	ApoB (g/L)	ApoAI/ ApoB
Bai Ku Yao								
AA	396	4.36 ± 0.85	1.02(0.65)	1.67 ± 0.40	2.58 ± 0.70	1.32 ± 0.32	0.85 ± 0.22	1.67 ± 0.71
AC/CC	230	4.29 ± 0.90	0.96(0.66)	1.64 ± 0.41	2.55 ± 0.75	1.29 ± 0.31	0.84 ± 0.22	1.65 ± 0.73
*F*	-	1.008	0.623	0.811	0.288	1.732	0.871	0.037
*P*	-	0.316	0.534	0.368	0.591	0.189	0.351	0.847
Male								
AA	191	4.35 ± 0.89	1.16(0.87)	1.72 ± 0.46	2.47 ± 0.72	1.39 ± 0.38	0.82 ± 0.21	1.86 ± 0.87
AC/CC	114	4.38 ± 1.02	1.02(0.74)	1.65 ± 0.46	2.57 ± 0.91	1.32 ± 0.35	0.85 ± 0.25	1.73 ± 0.92
*F*	-	0.087	0.297	1.728	1.176	2.781	1.100	1.688
*P*	-	0.768	0.766	0.190	0.279	0.096	0.295	0.195
Female								
AA	205	4.39 ± 0.81	0.94(0.54)	1.64 ± 0.34	2.68 ± 0.66	1.26 ± 0.25	0.89 ± 0.23	1.50 ± 0.45
AC/CC	116	4.19 ± 0.75	0.92(0.54)	1.62 ± 0.34	2.52 ± 0.56	1.24 ± 0.24	0.83 ± 0.19	1.56 ± 0.47
*F*	-	4.769	0.748	0.108	4.888	0.303	4.866	1.511
*P*	-	0.030	0.454	0.743	0.028	0.583	0.028	0.220
Han Chinese								
AA	472	4.73 ± 1.03	1.01(0.70)	1.89 ± 0.49	2.64 ± 0.78	1.41 ± 0.27	0.89 ± 0.23	1.69 ± 0.60
AC/CC	152	4.75 ± 1.00	1.00(0.64)	1.90 ± 0.52	2.60 ± 0.74	1.42 ± 0.29	0.89 ± 0.22	1.67 ± 0.50
*F*	-	0.041	0.307	0.100	0.263	0.164	0.005	0.084
*P*	-	0.839	0.759	0.751	0.608	0.685	0.946	0.772
Male								
AA	223	4.64 ± 1.05	1.04(0.75)	1.81 ± 0.48	2.59 ± 0.79	1.38 ± 0.28	0.88 ± 0.24	1.69 ± 0.69
AC/CC	81	4.69 ± 1.10	0.98(0.75)	1.84 ± 0.62	2.54 ± 0.81	1.39 ± 0.33	0.88 ± 0.22	1.64 ± 0.47
*F*	-	0.163	0.872	0.239	0.225	0.128	0.006	0.418
*P*	-	0.687	0.383	0.625	0.636	0.721	0.94	0.518
Female								
AA	249	4.81 ± 1.00	0.97(0.65)	1.96 ± 0.49	2.68 ± 0.78	1.45 ± 0.26	0.90 ± 0.21	1.69 ± 0.50
AC/CC	71	4.83 ± 0.87	1.07(0.58)	1.95 ± 0.39	2.69 ± 0.65	1.46 ± 0.24	0.91 ± 0.22	1.70 ± 0.53
*F*	-	0.031	0.344	0.007	0.007	0.017	0.060	0.016
*P*	-	0.861	0.731	0.935	0.932	0.896	0.806	0.898

TC, total cholesterol; TG, triglyceride; HDL-C, high-density lipoprotein cholesterol; LDL-C, low-density lipoprotein cholesterol; ApoAI, apolipoprotein AI; ApoB, apolipoprotein B.

### Relative factors for serum lipid parameters

Multiple linear regression analysis showed that the genotypes of rs1044925 polymorphism in the ACAT-1 gene were not correlated with any serum lipid parameters in the combined population of Bai Ku Yao and Han, in the Bai Ku Yao or in the Han population (Table 4). When multiple linear regression analysis was performed in the males and females in both ethnic groups; respectively, we found that the levels of TC, LDL-C and ApoB in Bai Ku Yao but not in Han were correlated with genotypes in females but not in males (*P *< 0.05 for all, Table 5). Serum lipid parameters were also correlated with sex, age, BMI, alcohol consumption, and blood pressure in both ethnic groups (Table 4 and 5).

**Table 4:** Correlative factors for serum lipid parameters between Bai Ku Yao and Han Chinese

Lipid parameter	Relative factor	Unstandardized coefficient	Std. error	Standardized coefficient	*t*	*P*
Bai plus Han						
TC	Body mass index	0.070	0.009	0.204	7.493	0.000
	Ethnic group	0.363	0.051	0.188	7.148	0.000
	Age	0.010	0.002	0.160	5.953	0.000
	Diastolic blood pressure	0.010	0.003	0.109	3.940	0.000
TG	Body mass index	0.070	0.012	0.165	5.742	0.000
	Alcohol consumption	0.185	0.074	0.076	2.478	0.013
	Sex	-0.247	0.074	-0.103	-3.355	0.001
	Diastolic blood pressure	0.008	0.003	0.066	2.286	0.022
HDL-C	Age	0.005	0.001	0.168	6.163	0.000
	Alcohol consumption	0.207	0.028	0.219	7.358	0.000
	Sex	0.127	0.028	0.135	4.566	0.000
	Body mass index	-0.015	0.005	-0.092	-3.334	0.001
	Diastolic blood pressure	0.003	0.001	0.057	2.024	0.043
	Ethnic group	0.247	0.025	0.264	9.979	0.000
LDL-C	Body mass index	0.059	0.007	0.224	8.004	0.000
	Age	0.007	0.001	0.143	5.136	0.000
	Alcohol consumption	-0.177	0.042	-0.117	-4.250	0.000
	Diastolic blood pressure	0.005	0.002	0.063	2.187	0.029
ApoAI	Age	0.004	0.001	0.194	7.110	0.000
	Alcohol consumption	0.143	0.018	0.233	7.849	0.000
	Ethnic group	0.120	0.016	0.198	7.479	0.000
	Diastolic blood pressure	0.002	0.001	0.069	2.519	0.012
	Sex	-0.037	0.018	-0.061	-2.068	0.039
ApoB	Body mass index	0.018	0.002	0.223	8.019	0.000
	Age	0.002	0.000	0.141	5.120	0.000
	Diastolic blood pressure	0.002	0.001	0.100	3.487	0.001
	Sex	0.032	0.012	0.071	2.613	0.009
	Ethnic group	0.034	0.012	0.076	2.852	0.004
ApoAI/ApoB	Alcohol consumption	0.227	0.037	0.172	6.219	0.000
	Body mass index	-0.034	0.006	-0.148	-5.360	0.000
Bai Ku Yao						
TC	Body mass index	0.087	0.014	0.239	6.190	0.000
	Age	0.008	0.002	0.138	3.570	0.000
TG	Alcohol consumption	0.218	0.065	0.155	3.366	0.001
	Body mass index	0.055	0.016	0.137	3.486	0.001
	Sex	-0.229	0.089	-0.119	-2.581	0.010
HDL-C	Alcohol consumption	0.117	0.023	0.198	5.099	0.000
	Age	0.004	0.001	0.152	3.905	0.000
LDL-C	Body mass index	0.077	0.012	0.255	6.587	0.000
	Age	0.006	0.002	0.124	3.215	0.001
	Alcohol consumption	-0.093	0.041	-0.089	-2.278	0.023
ApoAI	Alcohol consumption	0.140	0.018	0.300	7.951	0.000
	Age	0.003	0.001	0.151	3.999	0.000
ApoB	Body mass index	0.023	0.004	0.249	6.458	0.000
	Age	0.002	0.001	0.117	3.036	0.002
ApoAI/ApoB	Alcohol consumption	0.278	0.040	0.266	6.916	0.000
	Body mass index	-0.048	0.012	-0.159	-4.127	0.000
Han Chinese						
TC	Age	0.013	0.002	0.193	5.015	0.000
	Body mass index	0.055	0.012	0.173	4.410	0.000
	Diastolic blood pressure	0.017	0.004	0.173	4.285	0.000
	Sex	0.161	0.077	0.079	2.098	0.036
TG	Body mass index	0.071	0.018	0.164	4.005	0.000
	Alcohol consumption	0.309	0.090	0.135	3.448	0.001
	Diastolic blood pressure	0.011	0.005	0.084	2.033	0.042
HDL-C	Age	0.006	0.001	0.198	5.022	0.000
	Alcohol consumption	0.170	0.033	0.209	5.129	0.000
	Sex	0.212	0.040	0.213	5.258	0.000
	Body mass index	-0.020	0.006	-0.126	-3.172	0.002
	Diastolic blood pressure	0.005	0.002	0.099	2.429	0.015
LDL-C	Body mass index	0.053	0.009	0.221	5.726	0.000
	Age	0.009	0.002	0.190	4.858	0.000
	Alcohol consumption	-0.135	0.049	-0.107	-2.760	0.006
ApoAI	Age	0.004	0.001	0.251	6.467	0.000
	Alcohol consumption	0.086	0.018	0.189	4.721	0.000
	Sex	0.106	0.022	0.191	4.818	0.000
	Diastolic blood pressure	0.003	0.001	0.103	2.677	0.008
ApoB	Body mass index	0.015	0.003	0.218	5.547	0.000
	Age	0.003	0.001	0.195	5.069	0.000
	Diastolic blood pressure	0.003	0.001	0.119	2.979	0.003
ApoAI/ApoB	Body mass index	-0.027	0.007	-0.152	-3.847	0.000

TC, total cholesterol; TG, triglyceride; HDL-C, high-density lipoprotein cholesterol; LDL-C, low-density lipoprotein cholesterol; ApoAI, apolipoprotein AI; ApoB, apolipoprotein B

**Table 5:** Correlative factors for serum lipid parameters between males and females in both ethnic groups

Lipid parameter	Relative factor	Unstandardized coefficient	Std. error	Standardized coefficient	*t*	*P*
Bai Ku Yao						
Male						
TC	Body mass index	0.138	0.023	0.323	6.017	0.000
	Age	0.009	0.003	0.147	2.739	0.007
TG	Body mass index	0.100	0.031	0.185	3.256	0.001
	Alcohol consumption	0.308	0.153	0.115	2.015	0.045
HDL-C	Age	0.007	0.002	0.229	4.185	0.000
	Alcohol consumption	0.249	0.058	0.237	4.248	0.000
	Body mass index	-0.025	0.012	-0.118	-2.158	0.032
LDL-C	Body mass index	0.119	0.020	0.327	6.023	0.000
ApoAI	Alcohol consumption	0.226	0.046	0.271	4.969	0.000
	Age	0.005	0.001	0.203	3.716	0.000
Apo B	Body mass index	0.032	0.006	0.312	5.709	0.000
ApoAI/ApoB	Alcohol consumption	0.484	0.114	0.239	4.252	0.000
	Body mass index	-0.075	0.023	-0.184	-3.264	0.001
Female						
TC	Body mass index	0.044	0.017	0.139	2.511	0.013
	Genotype	-0.213	0.091	-0.129	-2.333	0.020
TG	Body mass index	0.031	0.014	0.124	2.241	0.026
LDL-C	Systolic blood pressure	0.005	0.002	0.128	2.326	0.021
	Body mass index	0.032	0.014	0.126	2.290	0.023
	Genotype	-0.151	0.072	-0.115	-2.086	0.038
ApoAI	Alcohol consumption	0.070	0.034	0.114	2.061	0.040
	Age	0.002	0.001	0.112	2.030	0.043
ApoB	Body mass index	0.013	0.005	0.152	2.777	0.006
	Systolic blood pressure	0.002	0.001	0.136	2.477	0.014
	Genotype	-0.052	0.025	-0.117	-2.128	0.034
ApoAI/ApoB	Diastolic blood pressure	-0.007	0.003	-0.132	-2.381	0.018
Han Chinese						
Male						
TC	Body mass index	0.065	0.018	0.206	3.552	0.000
	Age	0.012	0.004	0.179	3.229	0.001
	Diastolic blood pressure	0.013	0.006	0.138	2.347	0.020
TG	Body mass index	0.138	0.028	0.271	4.892	0.000
HDL-C	Alcohol consumption	0.308	0.055	0.297	5.587	0.000
	Age	0.009	0.002	0.267	4.962	0.000
	Body mass index	-0.022	0.008	-0.145	-2.771	0.006
LDL-C	Body mass index	0.047	0.013	0.204	3.612	0.000
	Age	0.006	0.003	0.132	2.341	0.020
ApoAI	Alcohol consumption	0.178	0.031	0.303	5.788	0.000
	Age	0.005	0.001	0.268	5.066	0.000
	Diastolic blood pressure	0.003	0.001	0.111	2.133	0.034
ApoB	Body mass index	0.018	0.004	0.262	4.797	0.000
	Age	0.003	0.001	0.199	3.645	0.000
ApoAI/ApoB	Alcohol consumption	0.183	0.073	0.143	2.515	0.012
	Body mass index	-0.035	0.011	-0.183	-3.123	0.002
	Systolic blood pressure	0.005	0.002	0.119	2.011	0.045
Female						
TC	Diastolic blood pressure	0.020	0.005	0.205	3.715	0.000
	Age	0.013	0.003	0.208	3.863	0.000
	Body mass index	0.045	0.017	0.143	2.662	0.008
TG	Diastolic blood pressure	0.016	0.005	0.160	2.884	0.004
HDL-C	Age	0.005	0.002	0.162	2.921	0.004
LDL-C	Body mass index	0.053	0.013	0.213	3.961	0.000
	Age	0.010	0.003	0.208	3.819	0.000
	Alcohol consumption	-0.319	0.095	-0.176	-3.346	0.001
	Diastolic blood pressure	0.009	0.004	0.124	2.242	0.026
ApoAI	Age	0.004	0.001	0.249	4.588	0.000
ApoB	Diastolic blood pressure	0.004	0.001	0.190	3.467	0.001
	Body mass index	0.014	0.004	0.196	3.654	0.000
	Age	0.003	0.001	0.191	3.559	0.000
ApoAI/ApoB	Body mass index	-0.028	0.009	-0.170	-3.076	0.002

TC, total cholesterol; TG, triglyceride; HDL-C, high-density lipoprotein cholesterol; LDL-C, low-density lipoprotein cholesterol; ApoAI, apolipoprotein AI; ApoB, apolipoprotein B

## Discussion

We showed that the levels of serum TC, HDL-C, ApoAI and ApoB were lower in Bai Ku Yao than in Han Chinese. There was no significant difference in the serum levels of TG, LDL-C and the ratio of ApoAI to ApoB between the two ethnic groups. It is well known that dyslipidemia is a multifactorial origin, including hereditary and acquired risk factors. Bai Ku Yao is a special and isolated subgroup of the Yao minority in China. There are about 30000 people of total Bai Ku Yao population who reside in two villages, Lihu and Baxu, Nandan County. Their ancestors began their migration from Hunan and Guizhou Province about Song Dynasty. Both Lihu and Baxu villages are typical infertile mountain area, usually it was called 30 percent soil with 70 percent rock. Thus, their income mostly comes from planting corn and paddy. Strict intra-ethnic marriages have been performed in this ethnic subgroup from time immemorial. Therefore, we believe that some hereditary characteristics and genotypes of lipid metabolism-related genes in this population may be different from those in Han Chinese.

In the present study, we showed that the frequency of C allele of rs1044925 in the ACAT-1 gene was higher in Bai Ku Yao (21.0%) than in Han Chinese (12.7%). The frequency of AC and CC genotypes was also higher in Bai Ku Yao than in Han. In a previous study in northern Han Chinese population, Zhao *et al. *[42] reported that there was no difference in the frequency of C allele between normal controls (9.7%) and Alzheimer's disease patients (9.3%). In another recent study in Han Chinese population, Li *et al. *[38] also found no significant difference in the frequency of C allele between normal controls (13.7%) and endogenous hypertriglyceridemia patients (15.3%). The allele frequency of the R526G variant in the ACAT-1 gene was not different between normolipidemic (67.6%) and hyperlipidemic Japanese subjects (63.3%). There was also no difference in the allele frequency of the -77G > A variant in the ACAT-1 gene between normolipidemic (50.3%) and hyperlipidemic subjects (51.5%) [39]. In the population of central and Southern Europe, however, the frequency of C allele of rs1044925 in the ACAT-1 gene was very high (35.4%) [40]. These results indicate that the prevalence of the C allele variation of rs1044925 in the ACAT-1 gene may have an ethnic specificity.

The association of rs1044925 polymorphism in the ACAT-1 gene and plasma or serum lipid profiles is not well documented. In a previous study in Japanese population, Ohta *et al. *[39] reported that the R526G variant in the ACAT-1 gene did not affect plasma lipid concentrations in subjects studied. However, among hyperlipidemic subjects, plasma HDL-C and ApoAI concentrations in subjects with -77G > A variant in the ACAT-1 gene were significantly higher than those in subjects without variant. In another recent study in Han Chinese population, Li *et al. *[38] found that control subjects with AA genotype had a higher serum mean concentrations of LDL-C and non-HDL-C when compared with those of C allele carriers (AC and CC genotype carriers), whereas hypertriglyceridemia subjects with AA genotype had a higher HDL-C level compared with those of C allele carriers. In the present study, we showed that the levels of TC, LDL-C and ApoB in Bai Ku Yao but not in Han were significant differences between the AA and AC/CC genotypes in females but not in males. The C allele carriers had lower serum TC, LDL-C and ApoB levels as compared with the C allele noncarriers. The levels of TC, LDL-C and ApoB in Bai Ku Yao but not in Han were also correlated with genotypes in females but not in males. These results suggest that the polymorphism of rs1044925 in the ACAT-1 gene is mainly associated with female serum TC, LDL-C and ApoB levels in the Bai Ku Yao population. The reason for this discrepancy between the two ethnic groups is unclear.

Several environmental factors were also found to affect serum lipid levels in this study. Serum lipid parameters were correlated with age, sex, alcohol consumption, BMI, and blood pressure. These data suggest that the environmental factors also play an important role in determining serum lipid levels in our populations [13,14]. The diet and lifestyle were different between the two ethnic groups. Corn was the staple food and rice, soy, buckwheat, sweet potato, and pumpkin products were the subsidiary foods in Bai Ku Yao. Approximately 90% of the beverages were corn wine and rum. The alcohol content is about 15% (v/v). They are also accustomed to drink hempseed soup and eat hempseed products. In contrast, rice was the staple food and corn, broomcorn, potato, and taro products were the subsidiary foods in Han. About 90% of the beverage was rice wine. The content of alcohol is about 30% (v/v). The staple and subsidiary foods are more favorable for serum lipid profiles in Bai Ku Yao than in Han. Corn contains abundant dietary fiber and plant protein [46]. Consumption of dietary fiber, specifically the soluble type, such as pectins and guar gum can decrease serum TC levels [47,48]. Corn fiber supplementation in men with hypercholesterolemia resulted in an additional lowering of serum TC, TG, and VLDL-C concentrations. Serum LDL-C and HDL-C concentrations were not significantly altered by corn fiber or wheat fiber supplementation [48]. Plant protein might promote the transportation and excretion of free cholesterol. Dietary soy protein has well-documented beneficial effects on serum lipid concentrations [49,50]. Buckwheat protein product has a potent hypocholesterolemic activity [51,52]. Low to high buckwheat seed and sprout meal can reduce serum TC levels. The high seed and sprout meals can also lower serum TG levels. The levels of serum LDL-C were significantly suppressed by all buckwheat meals. Serum HDL-C levels were increased, however, insignificantly. Nutraceutically more meaningful is that both LDL-C/HDL-C and TC/HDL-C ratios were significantly lowered [52]. Ingestion of 4 g/day caiapo (the extract of the white-skinned sweet potato Ipomoea batatas) for 6 weeks has been found to reduce plasma TC, LDL-C [53] and TG levels [54] in type 2 diabetic patients previously treated by diet alone. Adaramoye *et al. *[55] reported that supplemented diets containing 3% and 6% telfairia occidentalis (fluted pumpkin) in rats decreased plasma and postmitochondrial supernatant fraction (PMF) cholesterol levels by 20% and 30% and by 30% and 45%, respectively; decreased the cholesterol-induced increase in plasma and PMF LDL-C levels by 24% and 48% and by 28% and 52%, respectively; and decreased plasma and PMF lipid peroxidation by 24% and 20% and by 42% and 21%, respectively. Dietary hempseed is a rich source of polyunsaturated fatty acids (PUFAs). Hempseed-supplemented diet in animals displayed elevated plasma levels of PUFAs and a prominent enhancement in gamma-linolenic acid levels. When hempseed is added to a cholesterol-enriched diet, cholesterol-induced platelet aggregation returns to control levels [56,57]. This normalization may be partly due to increased levels of plasma gamma-linolenic acid [56]. In addition, several experimental and clinical studies have demonstrated that dietary hempseed or hempseed oil can decrease TC, TG and LDL-C levels [58-60], inhibit lipid peroxidation [61], and reduce atherogenic index [62].

## Conclusion

The present study shows that the frequency of C allele of rs1044925 in the ACAT-1 gene was higher in Bai Ku Yao than in Han Chinese. But only the levels of TC, LDL-C and ApoB in Bai Ku Yao but not in Han were different between the AA and AC/CC genotypes in females but not in males. The C allele carriers had lower serum TC, LDL-C and ApoB levels than the C allele noncarriers. The levels of TC, LDL-C and ApoB in Bai Ku Yao but not in Han were also correlated with genotypes in females but not in males. These results suggest that the polymorphism of rs1044925 in the ACAT-1 gene is mainly associated with female serum TC, LDL-C and ApoB levels in the Bai Ku Yao population.

## Competing interests

The authors declare that they have no competing interests.

## Authors' contributions

DFW participated in the design, undertook genotyping, and helped to draft the manuscript. RXY conceived the study, participated in the design, carried out the epidemiological survey, collected the samples, and drafted the manuscript. LHHA, XJH, XLC, LM, QL and TTY collaborated to the genotyping. JZW and SLP carried out the epidemiological survey, collected the samples, and helped to carry out the genotyping. All authors read and approved the final manuscript.

## References

[B1] Libby P The forgotten majority: unfinished business in cardiovascular risk reduction J Am Coll Cardiol 2005 46 1225 8 10.1016/j.jacc.2005.07.006 16198835

[B2] Breslow JL Cardiovascular disease burden increases, NIH funding decreases Nat Med 1997 3 600 1 10.1038/nm0697-600 9176478

[B3] Martin MJ Hulley SB Browner WS Kuller LH Wentworth D Serum cholesterol, blood pressure and mortality: implications from a cohort of 361,662 men Lancet 1986 2 933 6 10.1016/S0140-6736(86)90597-0 2877128

[B4] Hokanson JE Austin MA Plasma triglyceride level is a risk factor for cardiovascular disease independent of high-density lipoprotein cholesterol level: a meta-analysis of population-based prospective studies J Cardiovasc Risk 1996 3 213 9 10.1097/00043798-199604000-00014 8836866

[B5] März W Scharnagl H Winkler K Tiran A Nauck M Boehm BO Winkelmann BR Low-density lipoprotein triglycerides associated with low-grade systemic inflammation, adhesion molecules, and angiographic coronary artery disease: the Ludwigshafen Risk and Cardiovascular Health study Circulation 2004 110 3068 74 15505088 10.1161/01.CIR.0000146898.06923.80

[B6] Kwiterovich PO Jr Coresh J Smith HH Bachorik PS Derby CA Pearson TA Comparison of the plasma levels of apolipoproteins B and A-1, and other risk factors in men and women with premature coronary artery disease Am J Cardiol 1992 69 1015 21 10.1016/0002-9149(92)90856-T 1561971

[B7] Durrington PN Hunt L Ishola M Kane J Stephens WP Serum apolipoproteins AI and B and lipoproteins in middle aged men with and without previous myocardial infarction Br Heart J 1986 56 206 12 10.1136/hrt.56.3.206 3092846 PMC1236844

[B8] Boden WE High-density lipoprotein cholesterol as an independent risk factor in cardiovascular disease: Assessing the data from Framingham to the Veterans Affairs High-Density Lipoprotein Intervention Trail Am J Cardiol 2000 86 19L 22L 10.1016/S0002-9149(00)01464-8 11374850

[B9] Stamler J Daviglus ML Garside DB Dyer AR Greenland P Neaton JD Relationship of baseline serum cholesterol levels in 3 large cohorts of younger men to long-term coronary, cardiovascular, and all-cause mortality and to longevity JAMA 2000 284 311 8 10.1001/jama.284.3.311 10891962

[B10] LaRosa JC He J Vupputuri S Effect of statins on risk of coronary disease: a meta-analysis of randomized controlled trials JAMA 1999 282 2340 6 10.1001/jama.282.24.2340 10612322

[B11] Chen Z Peto R Collins R MacMahon S Lu J Li W Serum cholesterol concentration and coronary heart disease in population with low cholesterol concentrations BMJ 1991 303 276 82 10.1136/bmj.303.6797.276 1888927 PMC1670480

[B12] Eastern Stroke and Coronary Heart Disease Collaborative Research Group Blood pressure, cholesterol, and stroke in eastern Asia. Eastern Stroke and Coronary Heart Disease Collaborative Research Group Lancet 1998 352 1801 7 10.1016/S0140-6736(98)03454-0 9851379

[B13] Ruixing Y Qiming F Dezhai Y Shuquan L Weixiong L Shangling P Hai W Yongzhong Y Feng H Shuming Q Comparison of demography, diet, lifestyle, and serum lipid levels between the Guangxi Bai Ku Yao and Han populations J Lipid Res 2007 48 2673 81 10.1194/jlr.M700335-JLR200 17890682

[B14] Ruixing Y Dezhai Y Shuquan L Yuming C Hanjun Y Qiming F Shangling P Weixiong L Jing T Yiyang L Hyperlipidaemia and its risk factors in the Guangxi Bai Ku Yao and Han populations Public Health Nutr 2009 12 816 24 10.1017/S1368980008003273 18647432

[B15] Liu WY Yin RX Zhang L Cao XL Miao L Wu DF Aung LH Hu XJ Lin WX Yang DZ Association of the LIPG 584C > T polymorphism and serum lipid levels in the Guangxi Bai Ku Yao and Han populations Lipids Health Dis 2010 9 110 10.1186/1476-511X-9-110 20923576 PMC2976738

[B16] Zhang L Yin RX Liu WY Miao L Wu DF Aung LH Hu XJ Cao XL Wu JZ Pan SL Association of methylenetetrahydrofolate reductase C677T polymorphism and serum lipid levels in the Guangxi Bai Ku Yao and Han populations Lipids Health Dis 2010 9 123 10.1186/1476-511X-9-123 20977771 PMC2987990

[B17] Meng L Ruixing Y Yiyang L Xingjiang L Kela L Wanying L Lin Z Weixiong L Dezhai Y Shangling P Association of LIPC -250G > A polymorphism and several environmental factors with serum lipid levels in the Guangxi Bai Ku Yao and Han populations Lipids Health Dis 2010 9 28 10.1186/1476-511X-9-28 20222961 PMC2907871

[B18] Ruixing Y Yiyang L Meng L Kela L Xingjiang L Lin Z Wanying L Jinzhen W Dezhai Y Weixiong L Interactions of the apolipoprotein C-III 3238C > G polymorphism and alcohol consumption on serum triglyceride levels Lipids Health Dis 2010 9 86 20716347 10.1186/1476-511X-9-86 PMC2929234

[B19] Zhou Y Yin R Deng Y Li Y Wu J Interactions between alcohol intake and the polymorphism of rs708272 on serum high-density lipoprotein cholesterol levels in the Guangxi Hei Yi Zhuang population Alcohol 2008 42 583 91 10.1016/j.alcohol.2008.08.004 18835593

[B20] Heller DA de Faire U Pedersen NL Dahlén G McClearn GE Genetic and environmental influences on serum lipid levels in twins N Engl J Med 1993 328 1150 6 10.1056/NEJM199304223281603 8455681

[B21] Steinmetz J Boerwinkle E Gueguen R Visvikis S Henny J Siest G Multivariate genetic analysis of high density lipoprotein particles Atherosclerosis 1992 92 219 27 10.1016/0021-9150(92)90281-K 1385955

[B22] Pérusse L Rice T Després JP Bergeron J Province MA Gagnon J Leon AS Rao DC Skinner JS Wilmore JH Bouchard C Familial resemblance of plasma lipids, lipoproteins and postheparin lipoprotein and hepatic lipases in the HERITAGE Family Study Arterioscler Thromb Vasc Biol 1997 17 3263 9 9409321 10.1161/01.atv.17.11.3263

[B23] Yao Z McLeod RS Synthesis and secretion of hepatic apolipoprotein B-containing lipoproteins Biochim Biophys Acta 1994 1212 152 66 8180241 10.1016/0005-2760(94)90249-6

[B24] Sniderman AD Cianflone K Substrate delivery as a determinant of hepatic apoB secretion Arterioscler Thromb 1993 13 629 36 8485114 10.1161/01.atv.13.5.629

[B25] Thompson GR Naoumova RP Watts GF Role of cholesterol in regulating apolipoprotein B secretion by the liver J Lipid Res 1996 37 439 47 8728309

[B26] Katsuren K Fukuyama S Takata K Ohta T Effects of a new single-nucleotide polymorphism in the Acyl-CoA:cholesterol acyltransferase-2 gene on plasma lipids and apolipoproteins in patients with hyperlipidemia J Atheroscler Thromb 2003 10 32 6 12621162 10.5551/jat.10.32

[B27] Anderson RA Joyce C Davis M Reagan JW Clark M Shelness GS Rudel LL Identification of a form of acyl-CoA:cholesterol acyltransferase specific to liver and intestine in nonhuman primates J Biol Chem 1998 273 26747 54 10.1074/jbc.273.41.26747 9756918

[B28] Cases S Novak S Zheng YW Myers HM Lear SR Sande E Welch CB Lusis AJ Spencer TA Krause BR Erickson SK Farese RV Jr ACAT-2, a second mammalian acyl-CoA:cholesterol acyltransferase. Its cloning, expression, and characterization J Biol Chem 1998 273 26755 64 10.1074/jbc.273.41.26755 9756919

[B29] Joyce CW Shelness GS Davis MA Lee RG Skinner K Anderson RA Rudel LL ACAT1 and ACAT2 membrane topology segregates a serine residue essential for activity to opposite sides of the endoplasmic reticulum membrane Mol Biol Cell 2000 11 3675 87 11071899 10.1091/mbc.11.11.3675 PMC15029

[B30] Chang CC Huh HY Cadigan KM Chang TY Molecular cloning and functional expression of human acyl-coenzyme A:cholesterol acyltransferase cDNA in mutant Chinese hamster ovary cells J Biol Chem 1993 268 20747 55 8407899

[B31] Uelmen PJ Oka K Sullivan M Chang CC Chang TY Chan L Tissue-specific expression and cholesterol regulation of acylcoenzyme A:cholesterol acyltransferase (ACAT) in mice. Molecular cloning of mouse ACAT cDNA, chromosomal localization, and regulation of ACAT in vivo and in vitro J Biol Chem 1995 270 26192 201 10.1074/jbc.270.44.26192 7592824

[B32] Matsuda H Hakamata H Kawasaki T Sakashita N Miyazaki A Takahashi K Shichiri M Horiuchi S Molecular cloning, functional expression and tissue distribution of rat acyl-coenzyme A:cholesterol acyltransferase Biochim Biophys Acta 1998 1391 193 203 9555010 10.1016/s0005-2760(98)00007-1

[B33] Sakashita N Miyazaki A Takeya M Horiuchi S Chang CC Chang TY Takahashi K Localization of human acyl-coenzyme A: cholesterol acyltransferase-1 (ACAT-1) in macrophages and in various tissues Am J Pathol 2000 156 227 36 10623671 10.1016/S0002-9440(10)64723-2 PMC1868616

[B34] Oelkers P Behari A Cromley D Billheimer JT Sturley SL Characterization of two human genes encoding acyl coenzyme A:cholesterol acyltransferase-related enzymes J Biol Chem 1998 273 26765 71 10.1074/jbc.273.41.26765 9756920

[B35] Rudel LL Lee RG Cockman TL Acyl coenzyme A: cholesterol acyltransferase types 1 and 2: structure and function in atherosclerosis Curr Opin Lipidol 2001 12 121 7 10.1097/00041433-200104000-00005 11264983

[B36] Suckling KE Stange EF Role of acyl-CoA: cholesterol acyltransferase in cellular cholesterol metabolism J Lipid Res 1985 26 647 71 3897424

[B37] Smith JL Hardie IR Pillay SP de Jersey J Hepatic acyl-coenzyme A:cholesterol acyltransferase activity is decreased in patients with cholesterol gallstones J Lipid Res 1990 31 1993 2000 2086698

[B38] Li Q Bai H Fan P Analysis of acyl-coenzyme A: cholesterol acyltransferase 1 polymorphism in patients with endogenous hypertriglyceridemia in Chinese population Zhonghua Yi Xue Yi Chuan Xue Za Zhi 2008 25 206 10 18393248

[B39] Ohta T Takata K Katsuren K Fukuyama S The influence of the acyl-CoA:cholesterol acyltransferase-1 gene (-77G→A) polymorphisms on plasma lipid and apolipoprotein levels in normolipidemic and hyperlipidemic subjects Biochim Biophys Acta 2004 1682 56 62 15158756 10.1016/j.bbalip.2004.01.008

[B40] Wollmer MA Streffer JR Tsolaki M Grimaldi LM Lütjohann D Thal D von Bergmann K Nitsch RM Hock C Papassotiropoulos A Genetic association of acyl-coenzyme A: cholesterol acyltransferase with cerebrospinal fluid cholesterol levels, brain amyloid load, and risk for Alzheimer's disease Mol Psychiatry 2003 8 635 8 10.1038/sj.mp.4001296 12851640

[B41] People's Republic of China--United States Cardiovascular and Cardiopulmonary Epidemiology Research Group An epidemiological study of cardiovascular and cardiopulmonary disease risk factors in four populations in the People's Republic of China. Baseline report from the P.R.C.-U.S.A. Collaborative Study Circulation 1992 85 1083 96 1537106 10.1161/01.cir.85.3.1083

[B42] Zhao FG Wang YH Yang JF Ma QL Tang Z Dong XM Chan P Association between acyl-coenzyme A: cholesterol acyltransferase gene and risk for Alzheimer's disease in Chinese Neurosci Lett 2005 388 17 20 10.1016/j.neulet.2005.06.020 16043284

[B43] Ruixing Y Weixiong L Hanjun Y Dezhai Y Shuquan L Shangling P Qiming F Jinzhen W Jianting G Yaju D Diet, lifestyle, and blood pressure of the middle-aged and elderly in the Guangxi Bai Ku Yao and Han populations Am J Hypertens 2008 21 382 7 10.1038/ajh.2008.1 18369357

[B44] Ruixing Y Shangling P Shuquan L Dezhai Y Weixiong L Qiming F Yuming C Yaoheng H Yijiang Z Qinchen L Comparison of hypertension and its risk factors between the Guangxi Bai Ku Yao and Han populations Blood Press 2008 17 306 16 10.1080/08037050802589593 19043819

[B45] Cooperative Meta-analysis Group of China Obesity Task Force Predictive values of body mass index and waist circumference to risk factors of related diseases in Chinese adult population Chin J Epidemiol 2002 23 5 10 12015100

[B46] Dong W Ma X Zhang D Yu S Effect of maize embryo on delaying aging Food Sci 2002 23 95 7

[B47] Jenkins DJ Kendall CW Axelsen M Augustin LS Vusksan V Viscous and nonviscous fibres, nonabsorbable and low glycaemic index carbohydrates, blood lipids and coronary heart disease Curr Opin Lipidol 2000 11 49 56 10.1097/00041433-200002000-00008 10750694

[B48] Shane JM Walker PM Corn bran supplementation of a low-fat controlled diet lowers serum lipids in men with hypercholesterolemia J Am Diet Assoc 1995 95 40 5 10.1016/S0002-8223(95)00011-9 7798579

[B49] Weggemans RM Trautwein EA Relation between soy-associated isoflavones and LDL and HDL cholesterol concentrations in humans: a meta-analysis Eur J Clin Nutr 2003 57 940 6 10.1038/sj.ejcn.1601628 12879088

[B50] Zhan S Ho SC Meta-analysis of the effects of soy protein containing isoflavones on the lipid profile Am J Clin Nutr 2005 81 397 408 15699227 10.1093/ajcn.81.2.397

[B51] Tomotake H Shimaoka I Kayashita J Yokoyama F Nakajoh M Kato N Stronger suppression of plasma cholesterol and enhancement of the fecal excretion of steroids by a buckwheat protein product than by a soy protein isolate in rats fed on a cholesterol-free diet Biosci Biotechnol Biochem 2001 65 1412 4 10.1271/bbb.65.1412 11471745

[B52] Lin LY Peng CC Yang YL Peng RY Optimization of bioactive compounds in buckwheat sprouts and their effect on blood cholesterol in hamsters J Agric Food Chem 2008 56 1216 23 10.1021/jf072886x 18217700

[B53] Ludvik BH Mahdjoobian K Waldhaeusl W Hofer A Prager R Kautzky-Willer A Pacini G The effect of Ipomoea batatas (Caiapo) on glucose metabolism and serum cholesterol in patients with type 2 diabetes: a randomized study Diabetes Care 2002 25 239 40 10.2337/diacare.25.1.239 11772921

[B54] Ludvik B Hanefeld M Pacini G Improved metabolic control by Ipomoea batatas (Caiapo) is associated with increased adiponectin and decreased fibrinogen levels in type 2 diabetic subjects Diabetes Obes Metab 2008 10 586 92 10.1111/j.1463-1326.2007.00752.x 17645559

[B55] Adaramoye OA Achem J Akintayo OO Fafunso MA Hypolipidemic effect of Telfairia occidentalis (fluted pumpkin) in rats fed a cholesterol-rich diet J Med Food 2007 10 330 6 10.1089/jmf.2006.213 17651070

[B56] Prociuk MA Edel AL Richard MN Gavel NT Ander BP Dupasquier CM Pierce GN Cholesterol-induced stimulation of platelet aggregation is prevented by a hempseed-enriched diet Can J Physiol Pharmacol 2008 86 153 9 10.1139/Y08-011 18418423

[B57] Richard MN Ganguly R Steigerwald SN Al-Khalifa A Pierce GN Dietary hempseed reduces platelet aggregation J Thromb Haemost 2007 5 424 5 10.1111/j.1538-7836.2007.02327.x 17155962

[B58] Cen L Qin W Ye Y Effect of Canabis Sativa L on serum cholesterol level in rats J Guangxi Med Univ 1984 1 20 2

[B59] Qin W Cen L Ye Y The effect of some foods on serum cholesterol level in rats Acta Nutrimenta Sinica 1986 8 136 40

[B60] Schwab US Callaway J Erkkila AT Gynther J Uusitupa MI Jarvinen T Effects of hempseed and flaxseed oils on the profile of serum lipids, serum total and lipoprotein lipid concentrations and haemostatic factors Eur J Nutr 2006 45 470 7 10.1007/s00394-006-0621-z 17103080

[B61] Ren HY Sun HG Ma JZ Zhang Y Yi CR Wu MX Liu WL Li GL Experimental study on the effects of hemp fruit oil on serun lipid levels and lipid peroxidation Chin J Tradit Med Sci Technol 1997 4 200

[B62] Ren HY Sun HG Zhang Y Yi CR Wu MX Li GL Liu WL Lipid-lowering and antiatherosclerotic effects of hemp fruit oil in partridges Henan Tradit Chin Med 1998 18 294 5

